# Diagnostic value of lesion-targeted BALF immune biomarkers combined with T-SPOT.TB for smear-negative pulmonary tuberculosis

**DOI:** 10.1128/spectrum.03636-25

**Published:** 2026-09-01

**Authors:** Jie Ren, Yuning Huang, Xiujuan Bian, Di Wu, Xinxin Fan, Suqin Ben, Yawen Wang, Runqing Liu, Guogang Xie, Lihui Lin, Rong Tang, Hui Zhu, Min Zhang, Fengming Ding

**Affiliations:** 1 Department of Respiratory and Critical Care Medicine, Shanghai General Hospital, Shanghai Jiao Tong University School of Medicine 56694, Shanghai, China; 2 Department of Respiratory Medicine, Affiliated Renhe Hospital of Shanghai University (Renhe Hospital, Baoshan District) 664102 , Shanghai, China; 3 Department of Tuberculosis, Fujian Medical University Clinical Teaching Hospital, Fuzhou Pulmonary Hospital of Fujian Province 638885, Fuzhou, Fujian Province, China; 4 Department of Clinical Laboratory, Shanghai General Hospital, Shanghai Jiao Tong University School of Medicine 56694, Shanghai, China; Instituto de Salud Global de Barcelona, Barcelona, Spain

**Keywords:** bronchoalveolar lavage fluid, cytokine, effector T cells, prediction model, smear-negative pulmonary tuberculosis

## Abstract

Data on immune responses at local infection sites in smear-negative pulmonary tuberculosis (SNPT) remain limited. This study aimed to characterize lesion-targeted bronchoalveolar lavage fluid (BALF) cytokine and T-cell responses, and assess their diagnostic value for SNPT in clinically suspected patients. Patients were enrolled in a single-center derivation cohort between January 2021 and December 2023. Cytokine and lymphocyte subsets were measured in both peripheral blood and lesion-targeted BALF. Peripheral blood T-SPOT.TB assay and BALF targeted next-generation sequencing (tNGS) for *Mycobacterium tuberculosis* (Mtb) detection were performed. An immunological prediction model based on BALF immune biomarkers and peripheral blood T-SPOT.TB results was developed in the derivation cohort, and then externally validated in a three-center external validation cohort between June 2024 and May 2025. In the derivation cohort, 177 patients were included in the final analysis, including 69 SNPT patients and 108 non-SNPT patients. Stepwise logistic regression identified the BALF concentrations of IL-1β and IL-6, the BALF CD4/CD8 T-cell ratio, and the T-SPOT.TB result as independent predictors of SNPT, forming an immunological prediction model, with an area under the curve (AUC) of 0.856. Compared with T-SPOT.TB alone, the model increased specificity from 66.7% to 85.2% at an optimal predicted probability cut-off of 0.36. In the external validation cohort, another 177 suspected cases were analyzed, including 67 SNPT patients and 110 non-SNPT patients. The model achieved an AUC of 0.801, with a sensitivity of 71.6% and a specificity of 82.7% for SNPT prediction at the optimal cut-off. Parallel testing combining BALF tNGS and the prediction model yielded a sensitivity of 94.0% and a specificity of 78.0%, with an OR of 56.4 (95% CI, 18.7–170.8), exceeding that of BALF tNGS alone (OR, 18.7; 95% CI, 8.0–43.8). The measurement of BALF IL-1β and IL-6 concentrations and BALF CD4/CD8 T-cell ratio, combined with the T-SPOT.TB result, would facilitate the diagnosis of SNPT and benefit the effective initiation of anti-Mtb treatment.

**IMPORTANCE:**

Smear-negative pulmonary tuberculosis (SNPT) remains a critical diagnostic challenge. Our study identifies lesion-targeted immune biomarkers as independent predictors of SNPT and integrates these biomarkers with the peripheral interferon-γ release assay to develop an immunological prediction model. This model can improve diagnostic accuracy when used in parallel testing with the nucleic acid amplification assay. Our data indicate that the application of local immune biomarkers in the diagnosis of SNPT will benefit the effective initiation of anti-tuberculosis treatment and potentially reduce the spread of the disease.

## INTRODUCTION

Active pulmonary tuberculosis (PTB), a contagious airborne disease caused by the *Mycobacterium tuberculosis* (Mtb) complex, remains a major global cause of morbidity and mortality. Although co-infection with human immunodeficiency virus (HIV) markedly increases the risk and severity of PTB, non-HIV PTB cases remain a major global health concern, particularly in developing countries with high tuberculosis (TB) incidence (1). Sputum acid-fast bacillus (AFB) smear microscopy is the most widely used method for the rapid diagnosis of active PTB, but it lacks sensitivity, as smear-negative pulmonary tuberculosis (SNPT) has been reported to account for 30%–60% of active PTB cases (2, 3). SNPT is frequently underdiagnosed owing to low bacterial burden and atypical clinical and radiographic features. Although individual SNPT patients have approximately 22% of the infectious potential of smear-positive cases, their substantial numbers contribute to an estimated 17%–22% of community tuberculosis transmission (4, 5). In clinical practice, the non-SNPT category is heterogeneous, encompassing other active pulmonary diseases, as well as latent tuberculosis infection (LTBI), which is defined as a persistent immune response to Mtb antigens without clinically active disease (6). Differentiating SNPT from non-SNPT patients (including those with LTBI) is often challenging, leading to diagnostic delays of 60–90 days, progressive lung damage, and inappropriate treatment (7).

Efforts have focused on developing rapid diagnostics for SNPT to improve treatment and reduce transmission. Reliable, accessible, and easy-to-use modalities remain essential, as no single test fits all clinical scenarios (8). Current algorithms emphasize rapid molecular testing. The WHO recommends Xpert MTB/RIF and Xpert Ultra for the initial detection of TB and rifampicin resistance in sputum specimens (9). Although Xpert assays have shortened turnaround times, Xpert MTB/RIF still shows limited sensitivity in paucibacillary and smear-negative samples, whereas Xpert Ultra offers higher sensitivity in these scenarios (10). Targeted next-generation sequencing (tNGS) provides complementary information. Using cell-free DNA in bronchoalveolar lavage fluid (BALF), tNGS shows sensitivity comparable to Xpert MTB/RIF and detects non-rifampicin resistance mutations (11). Moreover, other studies support that tNGS offers higher sensitivity and broader resistance profiles than Xpert MTB/RIF (12–15). Importantly, tNGS can simultaneously detect both *Mycobacterium tuberculosis* complex and non-tuberculous mycobacteria (NTM), which aids in differentiating between the diseases of tuberculosis and NTM in clinically suspected patients (16). However, no molecular assay can fully exclude active PTB in smear-negative or paucibacillary disease, and trace detection of TB nucleic acid alone may cause false positivity and subsequent misdiagnosis (17, 18). Thus, the diagnostic strategy for SNPT still needs improvement.

Peripheral interferon-γ release assays like T-SPOT.TB (abbreviated as T-SPOT) rapidly detect Mtb exposure and guide empirical therapy in symptomatic patients with radiological suspicion of PTB (6). However, their diagnostic value for SNPT is limited, as peripheral blood lymphocytes poorly reflect the local immune profile of active disease (19). Thus, a positive T-SPOT in suspected SNPT indicates Mtb sensitization, not active PTB (3). Host immune profiling may provide additional information. Mtb-specific T-cell cytokines (IFN-γ, TNF-α, IL-2) help distinguish between Mtb infection states (20, 21); inflammatory cytokines (IL-1β, IL-6, IL-8) are involved in host defense (22–24); and immune-regulatory pathways (IL-10, IL-12, IL-17, IFN-α) are implicated in active TB (25–28). In severe PTB, the local cytokine profile may shift from Th1 to Th2 (29). However, most previous investigations were conducted using peripheral blood samples and therefore could not adequately reflect local pulmonary immune responses. Immune profiling in BALF is particularly relevant because it captures pulmonary immune responses at the infection site, where Mtb-host interactions occur directly (30). Although some studies have characterized BALF-based immune responses to Mtb (31, 32), they have not provided comprehensive local immune profiling, and evidence specifically focused on lesion-targeted BALF cytokine and lymphocyte subset responses in SNPT remains limited. Therefore, a systematic investigation of lesion-targeted BALF immune characteristics is warranted to evaluate their diagnostic potential for SNPT.

Based on evidence that local immune responses reflect active PTB pathogenesis, we hypothesize that lesion-targeted BALF biomarkers could independently predict SNPT and improve diagnostic accuracy when combined with interferon-γ release assays. In clinical practice, patients with suspected SNPT frequently undergo bronchoscopy for the differential diagnosis of other pulmonary conditions, a procedure that facilitates BALF collection from pulmonary lesions, particularly when sputum is inadequate. While BALF is primarily used for microbiological confirmation owing to its higher bacterial loads even in smear-negative cases, it also provides a valuable snapshot of local immune responses. This study therefore characterized cytokine and lymphocyte responses in such BALF and evaluated their combination with T-SPOT to diagnose SNPT in clinically suspected smear-negative patients.

## MATERIALS AND METHODS

### Study participants

This prospective study involved two independent cohorts of non-HIV adults (18–80 years) suspected of SNPT. The derivation cohort comprised patients enrolled from a single center between January 2021 and December 2023, whereas the external validation cohort consisted of patients from three centers between June 2024 and May 2025.

Inclusion criteria were as follows: (i) three consecutive negative sputum AFB smears; (ii) symptoms consistent with active PTB (fever, persistent cough ≥3 weeks, hemoptysis, chest pain, night sweats, or fatigue); (iii) radiological abnormalities (ground-glass opacities, consolidation, cavities, tree-in-bud signs, or bronchial wall thickening); (iv) bronchoscopy with bronchoalveolar lavage targeting the lesions. Exclusion criteria were (i) receipt of systemic immunosuppressive therapy (e.g., corticosteroids, TNF-α inhibitors, chemotherapy, or biologics) within the past 30 days; (ii) insufficient BALF cells for flow cytometry. To serve as positive controls for the validation study, five BALF AFB smear- and culture-positive PTB patients were additionally enrolled in the validation cohort. Demographic and clinical data were collected for all patients. The study protocol was approved by the ethics committees of the three participating centers (2024KS018, S-KY2025-11, and 2025-023KY-01). All patients provided written informed consent.

### Diagnostic procedures

The diagnostic workflow consisted of clinical assessment, chest imaging, sputum examination (including AFB smear microscopy and mycobacterial culture), peripheral blood T-SPOT assay, lesion-targeted bronchoscopy, BALF analysis (including smear microscopy, mycobacterial culture, and tNGS for Mtb detection), with final diagnostic classification based on clinical, radiological, microbiological, treatment-response, and follow-up evidence.

SNPT was defined as active PTB with three consecutive negative sputum AFB smear results and either culture-confirmed Mtb infection or a clinical diagnosis established after excluding alternative diseases and observing a lack of response to broad-spectrum antibiotics (excluding fluoroquinolones and aminoglycosides), with confirmation by symptom resolution and radiological improvement following 6 months of WHO-recommended anti-Mtb therapy. Patients without a confirmed diagnosis were excluded from the final analysis.

Non-SNPT patients were defined as disease-control patients who were initially suspected of SNPT but in whom active PTB was finally excluded, and an alternative respiratory diagnosis was established based on microbiological, pathological, radiological, clinical, and follow-up evidence. These patients included those with bacterial or fungal pneumonia, lung cancer, bronchiectasis, or other non-tuberculous lung diseases. All cases were reviewed by a multidisciplinary panel to ensure adherence to guideline criteria.

As a subgroup of non-SNPT, LTBI patients are characterized by a persistent immune response to Mtb antigens without clinically active PTB. In this study, they had positive T-SPOT results without microbiological evidence of TB, whereas other non-SNPT patients were T-SPOT negative, had neither microbiological evidence of Mtb nor known TB contact, and showed resolution of symptoms and pulmonary infiltrates without anti-TB treatment.

Notably, patients with NTM pulmonary disease were included in the non-SNPT control group to reflect real-world differential diagnosis and avoid spectrum bias, as clinicians frequently need to distinguish SNPT from other causes of pulmonary infiltrates, including NTM infection, in patients with negative sputum smears. These patients were diagnosed according to ATS/IDSA criteria, with microbiological confirmation on the basis of culture with species identification, supplemented by tNGS in selected cases. Sarcoidosis was also listed as one of the disease entities within the non-SNPT control group and was diagnosed based on compatible clinicoradiological features, histological evidence of non-caseating granulomas, and response to corticosteroid treatment.

### T-SPOT assay and the spot-forming cells

Peripheral blood T-SPOT assays (Oxford Immunotec, Marlborough, MA, USA) were performed according to the manufacturer’s protocol. Peripheral blood mononuclear cells (2.5 × 10⁵) were seeded into 96-well plates precoated with anti-IFN-γ antibody. Each patient had four wells: positive control, negative control, and two Mtb-specific antigen wells (early secreted antigenic target 6 [ESAT-6] and culture filtrate protein 10 [CFP-10]). Spot-forming cells were counted, with results considered positive if either antigen minus negative control ≥6 spots, and negative if both ≤5. Assays were repeated if control wells did not meet the manufacturer’s criteria.

### Sputum and BALF mycobacterial culture

Mycobacterial culture was performed on sputum and BALF specimens using the BACTEC MGIT 960 system with MGIT 7H9 medium according to standard microbiological procedures. Positive cultures were further identified by MPT64 antigen testing and PNB subculture or molecular methods to confirm mycobacterial species. All procedures involving suspected Mtb specimens were performed in an appropriate biosafety laboratory in accordance with institutional biosafety protocols.

### Bronchoalveolar lavage procedure and fluid processing for analysis

Bronchoalveolar lavage targeting lung segments identified by chest computed tomography was performed in a negative-pressure bronchoscopy room with standard airborne infection control precautions for patients suspected of pulmonary tuberculosis, using five 20 mL aliquots of 0.9% saline (total 100 mL; recovery >40%). For peripheral lesions, a bronchoscopic navigation system (DirectPath 1.0, Olympus, Tokyo, Japan) guided segmental bronchi selection. Recovered fluids (40–60 mL) were pooled; 10 mL was centrifuged at 200 × *g* for 5 min at 4°C for flow cytometry and cytokine analysis. The remaining 30–50 mL was used for Mtb-targeted tNGS (Hugobiotech, Beijing, China), differential cell counts, and cytology examination.

### BALF targeted next-generation sequencing

BALF tNGS was performed using the TB Pro assay (Hugobiotech, Beijing, China) on lesion-targeted BALF samples according to the testing laboratory’s validated workflow (33). This targeted sequencing assay was designed for *Mycobacterium tuberculosis* complex detection, NTM identification, and anti-tuberculosis drug-resistance-associated mutation profiling. Briefly, DNA was extracted and purified from BALF samples, quantified using a Qubit Fluorometer 4.0 (Thermo Fisher Scientific, MA, USA), and subjected to targeted amplification and sequencing-library preparation. Prepared libraries were sequenced on the NextSeq 550 platform (Illumina, San Diego, CA, USA). Sequencing data were processed using the laboratory’s bioinformatic pipeline, including read quality control, removal of host-derived reads, and alignment against Mycobacterium reference sequences and drug-resistance-associated mutation databases. The final report included pathogen identification, confidence level, pathogen-specific sequence counts, and detected drug-resistance-associated genes or mutations. Mtb positivity was determined according to the prespecified reporting criteria of the testing laboratory, and no additional investigator-defined read-count threshold was applied in this study. Quality control included assessment of nucleic acid quality, sequencing data quality, and internal controls.

### Measurement of cytokines and lymphocyte responses

Peripheral blood and BALF concentrations of IL-1β, IL-2, IL-4, IL-5, IL-6, IL-8, IL-10, IL-12, IL-17, IFN-α, IFN-γ, and TNF-α were determined using a panel kit (Raisecare, Qingdao, Shandong, China) by flow cytometry analysis according to the manufacturer’s instructions. These cytokines were selected to cover major immune pathways implicated in Mtb infection, including Th1-related responses (IFN-γ, IL-2, and IL-12), inflammatory and chemokine-mediated responses (IL-1β, IL-6, IL-8, and TNF-α), Th2/regulatory responses (IL-4, IL-5, and IL-10), Th17-related responses (IL-17), and type I interferon-related responses (IFN-α) (20, 22, 24–28). Samples were analyzed in duplicate, and the mean value was used for subsequent statistical analysis. Standard curves, blank wells, and assay quality-control samples were included in each plate, and assay results were accepted only when the standard curve (R² ≥ 0.99) and quality-control results met the manufacturer’s recommended criteria. To reduce potential batch effects, SNPT and non-SNPT samples were mixed across assay batches and run days, and the inter-batch coefficient of variation for all cytokines was below 12%.

Fresh peripheral blood and BALF samples were used for lymphocyte subset analysis without cryopreservation. PBMCs were isolated from peripheral blood by Ficoll-Paque density-gradient centrifugation, whereas BALF cells were pelleted, washed, and resuspended in PBS before staining. Lymphocytes were analyzed with a Beckman Coulter EPICS XL flow cytometer using monoclonal antibodies (Beckman Coulter, Inc., Miami, FL, USA) for T-cell subsets, B-cells, and NK cells, which represent major adaptive and innate-like lymphocyte compartments in Mtb-related cellular immunity (20, 21, 32). Additional markers were used to assess activation (HLA-DR/CD69), regulatory phenotypes (CD3^+^CD25^+^CD127^−^), memory/naive differentiation (CD45RO/CD45RA), costimulatory phenotype (CD8^+^CD28^+^), and helper-associated phenotype (CD4^+^CD29^+^) (25, 34).

At least 10,000 events within the lymphocyte gate were acquired for peripheral blood samples. BALF samples were considered evaluable if ≥2,000 lymphocyte events were acquired; otherwise, they were excluded from flow-cytometric analysis. Data were analyzed with FlowJo v10 (BD Biosciences). Lymphocytes were gated by forward/side scatter, followed by singlet and lineage-marker gating. Subset frequencies were calculated relative to parental gates. Representative gating plots are provided in Fig. S1.

### Statistical analysis

Statistical analyses were performed using SPSS Statistics version 26.0 (IBM Corp., Armonk, NY, USA), R software version 4.5.0 (R Foundation for Statistical Computing, Vienna, Austria), and GraphPad Prism software version 9 (GraphPad, San Diego, CA, USA). Quantitative variables were expressed as mean (SD) or median (interquartile range [IQR]) according to distribution. Paired biomarkers between peripheral blood and BALF were compared using the Wilcoxon signed-rank test; comparisons between independent groups used the Mann-Whitney *U* test. Categorical variables were analyzed with chi-square tests (or Fisher’s exact tests when frequencies or counts were <5). Receiver operating characteristic (ROC) analyses evaluated diagnostic performance, with the area under the curve (AUC) and optimal cut-offs determined by Youden’s index. Stepwise logistic regression identified independent SNPT predictors, incorporating peripheral blood T-SPOT results and BALF biomarkers, which were then used to construct a prediction model. The entry and removal criteria for the stepwise procedure were *P* < 0.05 and *P* > 0.10, respectively. Multicollinearity among variables retained in the final model was assessed using variance inflation factors (VIF < 2.5 for all final variables). The diagnostic performance of tNGS, T-SPOT, the prediction model, and their combined parallel testing was systematically compared. External validation was performed in an independent cohort. Spearman’s rank correlation assessed associations between nonparametric variables. Given the exploratory nature of this analysis aimed at identifying candidate immune markers, we did not apply formal correction for multiple comparisons. As a result, the associations identified should be interpreted with caution; their robustness is supported by subsequent validation in an independent cohort. To reduce the likelihood of false-positive findings, a conservative significance threshold of *P* < 0.01 was adopted for these group comparisons.

## RESULTS

### Baseline characteristics and diagnostic classification of the derivation and validation cohorts

**Fig 1:**
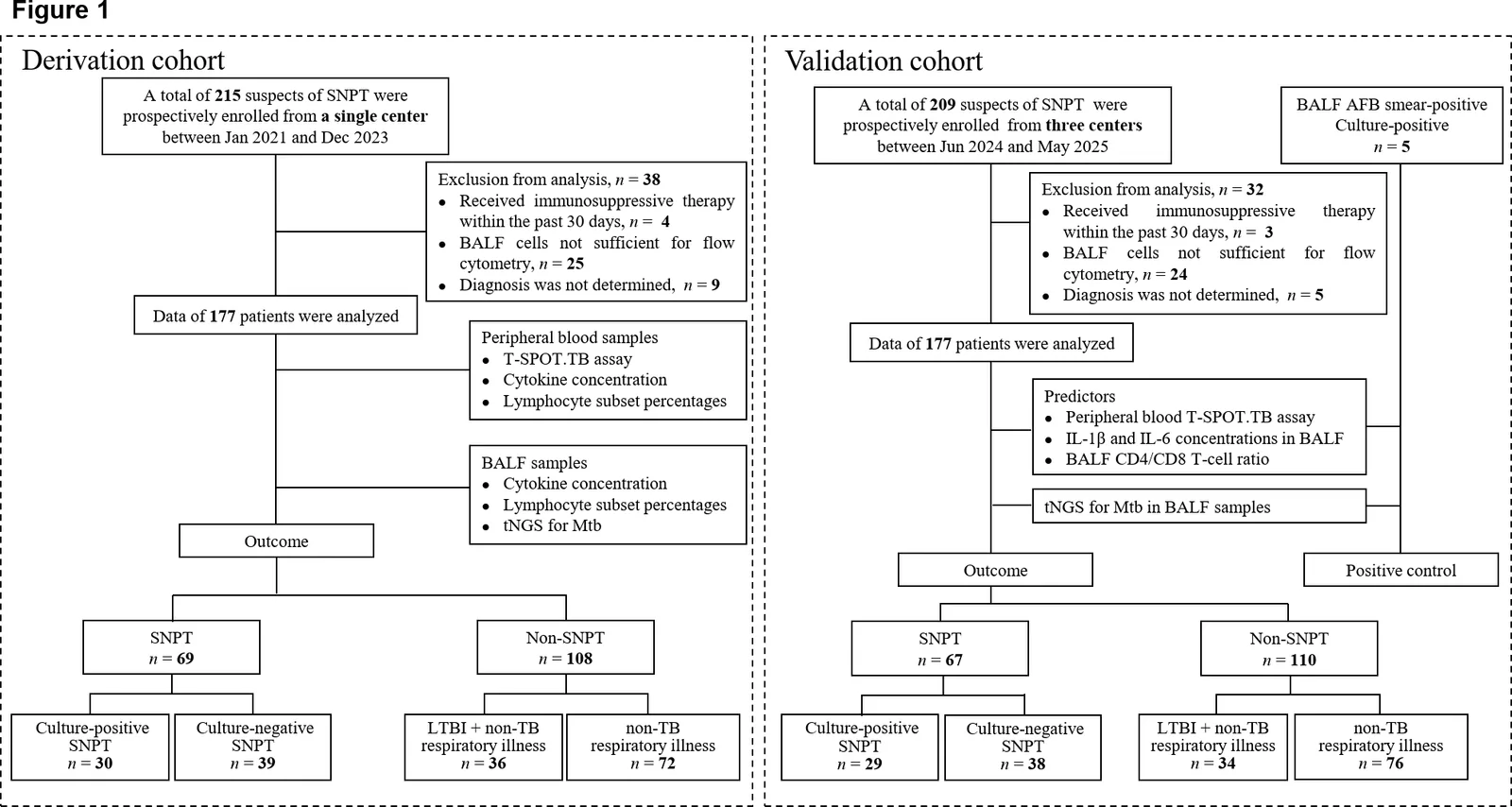
The flowchart of patient selection for the derivation and validation cohorts. SNPT, smear-negative pulmonary tuberculosis; LTBI, latent tuberculosis infection; BALF, bronchoalveolar lavage fluid; IFN-γ, interferon-gamma; tNGS, targeted next-generation sequencing; AFB, acid-fast bacilli.

Of the 215 patients initially suspected of SNPT in the derivation cohort, 38 were excluded due to systemic immunosuppressive therapy within the past 30 days (*n* = 4), insufficient BALF cells available for flow cytometry (*n* = 25), or an undetermined final diagnosis (*n* = 9), leaving 177 patients for the final analysis. Among these, 69 (39.0%) were diagnosed with SNPT (30 culture-positive, 39 clinically diagnosed), and 108 had confirmed alternative respiratory illnesses, including bacterial pneumonia (54), fungal pneumonia (5), non-tuberculous mycobacterial infection (2), lung cancer (15), sarcoidosis (11), bronchiectasis (7), idiopathic pulmonary fibrosis (5), cryptogenic organizing pneumonia (3), bronchiolitis (3), and prior PTB without reactivation (3). The study flowchart is shown in Fig. 1, and baseline characteristics are summarized in Table 1. T-SPOT was positive in 49/69 (71.0%) SNPT patients and 36/108 (33.3%) non-SNPT patients (diagnostic OR 4.9; 95% CI, 2.5–9.4; *P* < 0.001). In culture-confirmed SNPT cases, T-SPOT sensitivity and specificity were 63.3% and 66.7%, with no significant difference between culture-positive and culture-negative SNPT cases (*P* = 0.217). ESAT-6 and CFP-10 spot-forming cell (SFC) counts were 9 (4–29) and 6 (1–32) in SNPT, and 10 (4–20) and 7 (3–15) in LTBI patients (*P* = 0.886 and *P* = 0.727, respectively).

**TABLE 1:** Demographic and clinical characteristics of patients in the derivation cohort^*a*^^,^^*b*^^*,*^^*c*^

	Non-SNPT		SNPT		Non-SNPT vs SNPT	Culture-negative SNPT vs culture-positive SNPT
	Non-TB respiratory illness *n* = 72	LTBI + non-TB respiratory illness *n* = 36	Culture-negative SNPT *n* = 39	Culture-positive SNPT *n* = 30	*P* value	*P* value
Gender, male/female (% of male)	35/37 (48.6)	21/15 (58.3)	20/19 (51.3)	21/9 (70.0)	0.324	0.116
Age in years, mean (SD)	56.1 (16.1)	61.2 (12.2)	52.3 (18.5)	55.0 (18.5)	0.088	0.878
BMI, kg/m^2^ (SD)	22.8 (2.9)	23.3 (3.2)	21.8 (2.7)	21.9 (2.9)	0.012	0.712
Diabetes mellitus, no. (%)	11 (15.3)	1 (2.8)	7 (17.9)	7 (23.3)	0.092	0.581
Symptoms						
Fever, no. (%)	3 (4.2)	4 (11.1)	3 (7.7)	8 (26.7)	0.042	0.071
Cough, no. (%)	47 (65.3)	21 (58.3)	30 (76.9)	24 (80.0)	0.032	0.759
Coughing up blood or mucus, no. (%)	28 (38.9)	12 (33.3)	17 (43.6)	12 (40.0)	0.507	0.765
Chest pain, no. (%)	8 (11.1)	5 (13.9)	8 (20.5)	3 (10.0)	0.459	0.395
Night sweats, no. (%)	0 (0.0)	1 (2.8)	2 (5.1)	2 (6.7)	0.076	0.786
Tiredness, no. (%)	3 (4.2)	0 (0.0)	5 (12.8)	4 (13.3)	0.012	0.950
Peripheral blood cell count (10^9^/L)						
Leukocyte number, median (IQR)	6.3 (5.2–8.5)	6.3 (5.1–7.4)	6.6 (5.3–7.5)	6.2 (5.0–7.6)	0.983	0.747
Neutrophil number, median (IQR)	3.8 (2.6–4.9)	3.7 (2.9–4.2)	4.2 (3.0–5.3)	3.9 (3.1–5.1)	0.279	0.846
Lymphocyte number, median (IQR)	1.8 (1.3–2.4)	1.9 (1.3–2.4)	1.7 (1.3–1.9)	1.5 (0.8–1.9)	**0.005**	0.226
Monocyte number, median (IQR)	0.4 (0.3–0.7)	0.4 (0.3–0.5)	0.5 (0.4–0.6)	0.4 (0.3–0.5)	0.883	0.304
T-SPOT.TB						
Positivity rates, no. (%)	0 (0.0)	36 (100.0)	30 (76.9)	19 (63.3)	**<0.001**	0.217
SFC for ESAT-6 (/10⁶ PBMCs), median (IQR)	0.0 (0.0–0.0)	9.5 (4.3–20.0)	12.0 (6.0–30.0)	7.0 (2.5–22.5)	**<0.001**	0.157
SFC for CFP-10 (/10⁶ PBMCs), median (IQR)	0.0 (0.0–0.0)	7.0 (3.0–14.8)	9.0 (1.0–23.0)	5.0 (0.0–41.5)	**<0.001**	0.773
BALF cell count (10^6^/L)						
Nucleated cell number, median (IQR)	115.0 (48.5–238.8)	61 (17.0–145.0)	100.0 (43.0–280.0)	495.0 (267.5–1,350.0)	**0.001**	**<0.001**
Neutrophil number, median (IQR)	4.5 (1.3–18.1)	2.7 (0.8–18.8)	6.0 (1.7–43.2)	234.8 (8.9–1,215.0)	**<0.001**	**0.001**
Lymphocyte number, median (IQR)	7.8 (1.1–25.7)	5.0 (1.7–8.7)	12.6 (2.6–48.0)	15.7 (7.6–53.4)	**0.002**	0.351
Macrophage number, median (IQR)	58.6 (16.4–126.8)	28.4 (5.6–83.2)	54.6 (13.0–100.8)	113.7 (50.6–253.4)	0.053	**0.007**
Radiological manifestation						
Extent of lung lesions						
<25%, no. (%)	61 (84.7)	30 (83.3)	21 (53.8)	15 (50.0)	**<0.001**	0.751
25%–50%, no. (%)	10 (13.9)	6 (16.7)	18 (46.2)	15 (50.0)	**<0.001**	0.751
>50%, no. (%)	1 (1.4)	0 (0.0)	0 (0.0)	0 (0.0)	ND	ND
Cavity, no. (%)	1 (1.4)	0 (0.0)	4 (10.3)	4 (13.3)	**0.006**	0.987
Bilateral disease, no. (%)	44 (61.1)	17 (47.2)	24 (61.5)	18 (60.0)	0.612	0.897
Positivity rates of culture for Mtb, no. (%)	0 (0.0)	0 (0.0)	0 (0.0)	30 (100.0)	ND	ND
Positivity rates of tNGS for Mtb, no. (%)	5 (6.9)	3 (8.3)	14 (35.9)	26 (86.7)	**<0.001**	**<0.001**

^*a*^ The data were compared by chi-square test, except that age was compared by unpaired *t*-test, and BMI, peripheral blood cell analysis, and BALF cell analysis were compared by Mann-Whitney *U* test.

^*b*^ Statistically significant differences (*P* < 0.01) are indicated by bold text.

^*c*^ LTBI, latent tuberculosis infection; SNPT, smear-negative pulmonary tuberculosis; SD, standard deviation; BMI, body mass index; IQR, interquartile range; CFP-10, culture filtrate protein 10; ESAT-6, early secreted antigenic target 6; PBMC, peripheral blood mononuclear cells; Mtb, *Mycobacterium tuberculosis*; BALF, bronchoalveolar lavage fluid; tNGS, targeted next-generation sequencing; ND, not determined.

In the external validation cohort, of 209 suspected patients, 32 were excluded due to systemic immunosuppressive therapy within the past 30 days (*n* = 3), insufficient BALF cells available for flow cytometry (*n* = 24), or an undetermined final diagnosis (*n* = 5), leaving 177 for validation. Of these, 67 were diagnosed with SNPT (29 culture-positive, 38 clinically diagnosed). Mtb-specific sequences were detected by tNGS in 44/67 (65.7%) SNPT patients. The 110 non-SNPT patients had confirmed alternative diagnoses: bacterial pneumonia (42), fungal pneumonia (18), non-tuberculous mycobacterial infection (3), lung cancer (12), cryptogenic organizing pneumonia (8), bronchiectasis (7), idiopathic pulmonary fibrosis (6), sarcoidosis (7), bronchiolitis (5), and prior PTB without reactivation (2). The flowchart and main outcomes for the external cohort are also shown in Fig. 1, with baseline characteristics in Table S1.

### BALF concentration of cytokines in the derivation cohort

**Fig 2:**
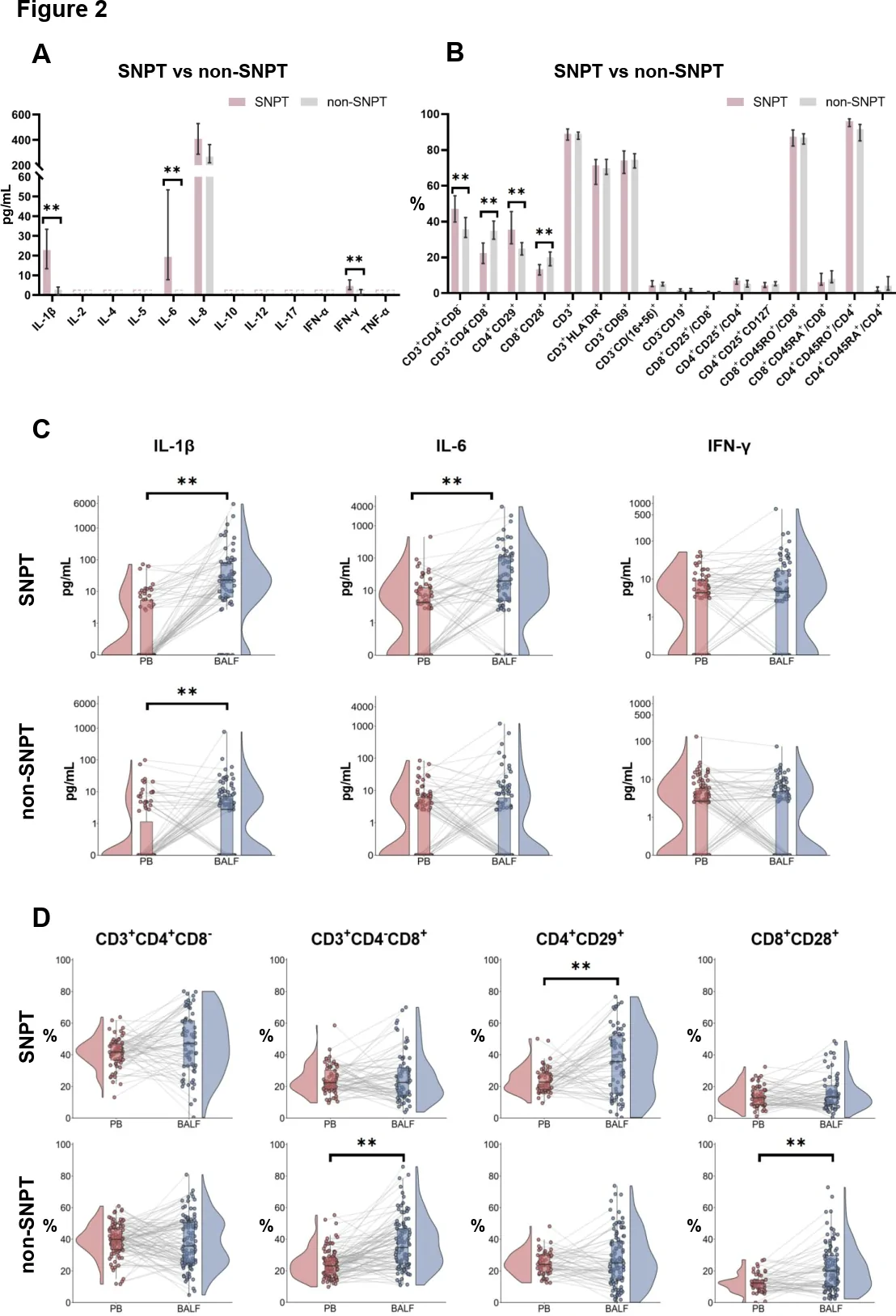
Profiles of cytokine levels and lymphocyte subset percentages in lesion-targeted BALF from SNPT-suspected patients in the derivation cohort. (**A**) BALF cytokine concentrations were compared between SNPT and non-SNPT. (**B**) BALF lymphocyte subset percentages were compared between SNPT and non-SNPT patients. All immune-cell frequencies were calculated relative to their corresponding parental gates. Data in panels** A **and **B** were compared using Mann-Whitney *U* tests. (**C**) Comparison of IL-1β, IL-6, and IFN-γ concentrations between paired peripheral blood and BALF samples in SNPT and non-SNPT patients. (**D**) Comparison of total CD4 cells, total CD8 cells, CD4^+^CD29^+^ cells, and CD8^+^CD28^+^ cells between paired peripheral blood and BALF samples in SNPT and non-SNPT patients. Data in panels **C** and **D** were compared using paired Wilcoxon tests. Statistically significant differences are depicted as **P < 0.01. PB, peripheral blood; BALF, bronchoalveolar lavage fluid; SNPT, smear-negative pulmonary tuberculosis; IL, interleukin; IFN-γ, interferon-gamma.

Of the 12 cytokines measured in the BALF, concentrations of IL-1β, IL-6, and IFN-γ were significantly higher in SNPT patients than in non-SNPT patients (Fig. 2A), with no corresponding differences in blood. In SNPT patients, concentrations of IL-1β and IL-6 were clearly elevated in BALF compared with peripheral blood. The BALF-to-blood ratios of IL-1β and IL-6 were 4.4 (1.3–10.5) and 4.5 (1.1–17.0), respectively, indicating a localized accumulation of Mtb-related cytokines at the site of infection in active PTB (Fig. 2C). However, in non-SNPT patients, only IL-1β was increased in BALF.

### BALF lymphocyte subset responses in the derivation cohort

For BALF lymphocyte subsets, SNPT patients had significantly higher percentages of total CD4 cells (*P* = 0.002) and significantly lower percentages of total CD8 cells (*P* < 0.001) than non-SNPT patients, resulting in a higher CD4/CD8 T-cell ratio for SNPT patients (*P* < 0.001, Fig. 2B). SNPT patients also showed significantly higher CD4^+^CD29^+^ (*P* = 0.004) and significantly lower CD8^+^CD28^+^ percentages (*P* = 0.009) in BALF. The above differences were absent in peripheral blood. Comparing lymphocyte subset percentages in BALF with those in peripheral blood, SNPT patients had significantly higher percentages of CD4^+^CD29^+^ cells in BALF, with a BALF-to-blood ratio of 1.5 (0.8–2.5), while non-SNPT patients had significantly higher percentages of CD8^+^CD28^+^ cells in BALF, with a BALF-to-blood ratio of 1.4 (0.7–2.4) (Fig. 2D). Correlation analysis of BALF lymphocyte subset percentages revealed that the CD4/CD8 T-cell ratio was positively correlated with the percentages of CD4^+^CD29^+^ cells (*rs* = 0.584, *P* < 0.001), and negatively correlated with the percentages of CD8^+^CD28^+^ cells (*rs* = −0.524, *P* < 0.001).

### Immunological modeling for predicting SNPT with BALF biomarkers and peripheral blood T-SPOT results

**Fig 3:**
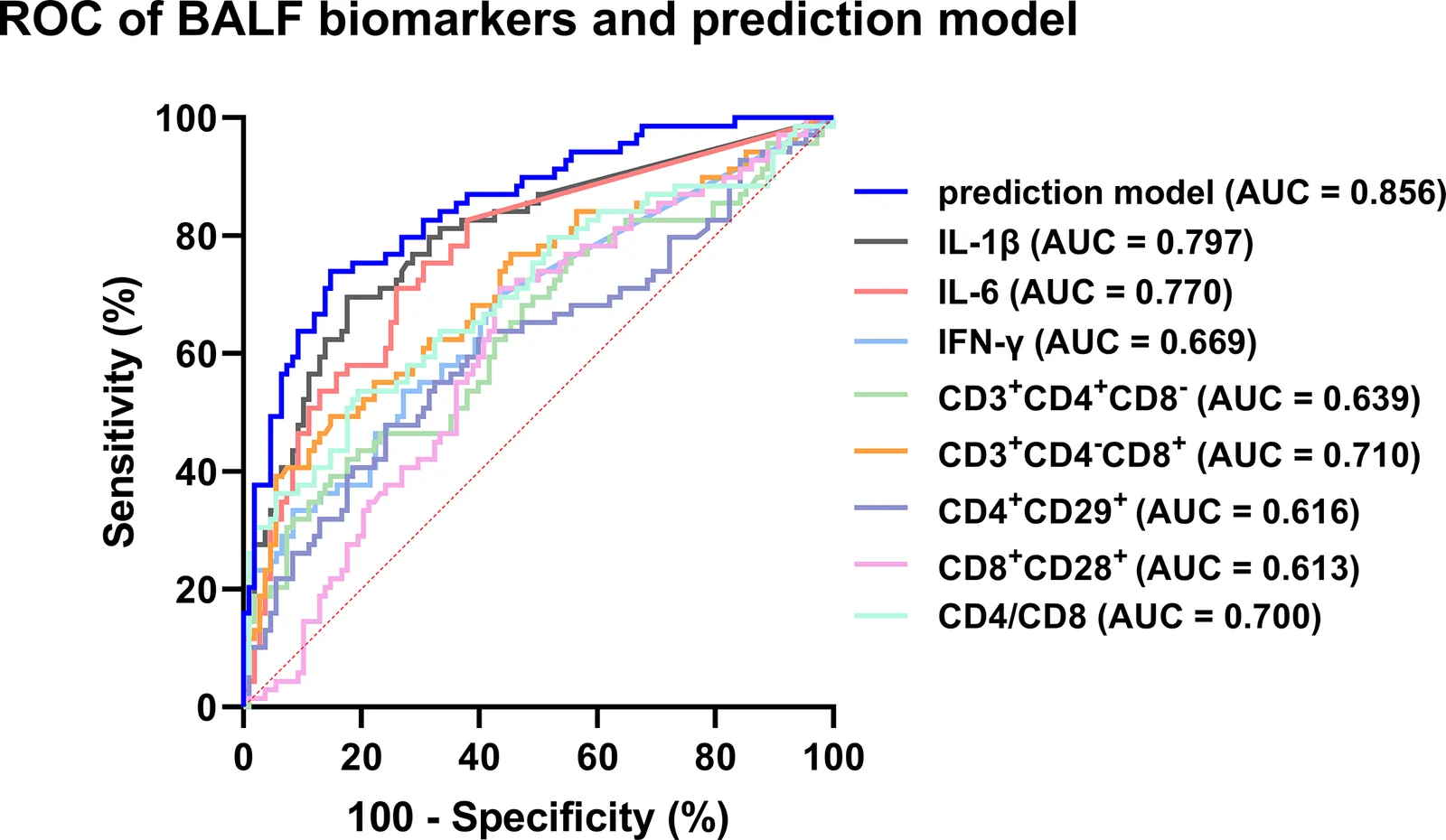
Receiver operating characteristic curves of different biomarkers and the prediction model for SNPT in the derivation cohort. Curves depict the BALF cytokine concentrations (IL-1β, IL-6, and IFN-γ), BALF percentages of lymphocyte subsets (total CD4 cells, total CD8 cells, CD4^+^CD29^+^ cells, and CD8^+^CD28^+^ cells), BALF CD4/CD8 T-cell ratio, and the combined prediction model. The corresponding AUC values for each biomarker and the prediction model are shown in the figure. Prediction model: ln[P/(1 – P)] = −2.236 + 0.038 × SFC_ESAT-6_ + 0.033 × SFC_CFP-10_ + 0.005 × C_IL-1β_ + 0.001 × C_IL-6_ + 0.481 × R_CD4/CD8_. P, predicted probability of SNPT; SFC_ESAT-6_, peripheral blood SFCs of ESAT-6; SFC_CFP-10_, peripheral blood SFCs of CFP-10; C_IL-1β_, BALF concentration of IL-1β (pg/mL); C_IL-6_, BALF concentration of IL-6 (pg/mL); R_CD4/CD8_, BALF ratio of CD4/CD8 T-cells; IL, interleukin; IFN-γ, interferon-gamma; SNPT, smear-negative pulmonary tuberculosis; ROC, receiver operating characteristic; AUC, area under the receiver operating characteristic curve.

ROC curves were calculated for BALF biomarkers differing between SNPT and non-SNPT patients, including concentrations of the cytokines (IL-1β, IL-6, IFN-γ), lymphocyte subset percentages (total CD4 cells, total CD8 cells, CD4^+^CD29^+^ cells, CD8^+^CD28^+^ cells), and CD4/CD8 T-cell ratio (Fig. 3). Optimal cut-offs were determined by Youden’s index. Stepwise logistic regression with these biomarkers and the peripheral blood T-SPOT identified four independent predictors: OR = 5.8 (95% CI, 2.4–13.8) for IL-1β concentration, OR = 4.8 (95% CI, 2.0–11.4) for IL-6 concentration, OR = 5.6 (95% CI, 2.7–15.8) for CD4/CD8 T-cell ratio, and OR = 3.9 (95% CI, 1.7–9.0) for T-SPOT result. For the final model, the binary T-SPOT result was replaced by quantitative ESAT-6 and CFP-10 SFC responses to retain antigen-specific information. No significant multicollinearity was observed among the predictors retained in the final model, with all VIF values below 2.5. The resulting logistic regression model for SNPT prediction was as follows:

ln[P/(1 – P)] = −2.236 + 0.038 × SFC_ESAT-6_ + 0.033 × SFC_CFP-10_ + 0.005 × C_IL-1β_ + 0.001 × C_IL-6_ + 0.481 × R_CD4/CD8^.^_

P = predicted probability of SNPT; SFC_ESAT-6_ = peripheral blood SFCs of ESAT-6; SFC_CFP-10_ = peripheral blood SFCs of CFP-10; C_IL-1β_ = BALF concentration of IL-1β (pg/mL); C_IL-6_ = BALF concentration of IL-6 (pg/mL); R_CD4/CD8_ = BALF ratio of CD4/CD8 T-cells.

The SNPT predicted probability from the combination model showed an ROC AUC of 0.856 (95% CI, 0.797–0.914). Further subgroup analysis showed that the AUC for culture-positive SNPT patients was 0.817 (95% CI, 0.729–0.905), while the AUC for culture-negative SNPT patients was 0.875 (95% CI, 0.811–0.939), with no significant difference between the two groups (*P* = 0.370). According to the maximized Youden’s index, the optimal cut-off value of predicted probability was determined as 0.36. Using this cut-off, the sensitivity and specificity of this model for SNPT prediction were 73.9% (95% CI, 62.5% to 82.8%) and 85.2% (95% CI, 77.3% to 90.7%).

### Diagnostic value of the prediction model in the derivation cohort

Mtb-targeted tNGS was performed on BALF in 165 of 177 patients (69 SNPT, 96 non-SNPT). Mtb-specific DNA sequences were detected in 40/69 (58.0%) SNPT patients, and in 8/96 (8.3%) non-SNPT patients, corresponding to a sensitivity of 58.0% and a specificity of 91.7% (OR, 15.2; 95% CI, 6.4–36.1; *P* < 0.001). In this population, using the optimal cut-off of predicted probability ≥0.36, the prediction model identified 51/69 (73.9%) SNPT patients and 13/96 (13.5%) non-SNPT patients, corresponding to a sensitivity of 73.9% and a specificity of 86.5% (OR, 18.1; 95% CI, 8.2–40.0; *P* < 0.001). Comparison of assay agreement between the prediction model and tNGS yielded a Cohen’s kappa of 0.610 (95% CI, 0.49–0.73), indicating substantial agreement according to Landis and Koch’s criteria. Parallel testing combining the prediction model and tNGS increased sensitivity to 88.4% while maintaining 79.2% specificity (OR, 29.0; 95% CI, 11.9–70.3), reducing the missed diagnosis rate from 42.0% with tNGS alone to 11.6%, thus avoiding missed diagnoses in approximately one-third of SNPT patients. These diagnostic performance metrics are summarized in Table 2.

**TABLE 2:** Comparison of methods for the detection of active tuberculosis^*a*^^,^^*b*^

Parameter	Sensitivity	Specificity	Positive predictive value	Negative predictive value	Positive likelihood ratio	Negative likelihood ratio	Area under ROC curve	Odds ratio
The derivation cohort								
T-SPOT.TB (positive)	0.71	0.67	0.58	0.78	2.13	0.43	0.688	4.9 (95% CI, 2.5–9.4)
Prediction model (probability ≥ 0.36)	0.74	0.87	0.80	0.82	5.46	0.30	0.856	18.1 (95% CI, 8.2–40.0)
BALF tNGS (positive)	0.58	0.92	0.83	0.75	6.96	0.46	0.748	15.2 (95% CI, 6.4–36.1)
Parallel testing								
BALF tNGS with T-SPOT.TB	0.87	0.61	0.62	0.87	2.26	0.21	0.742	10.6 (95% CI, 4.7–24.0)
BALF tNGS with prediction model	0.88	0.79	0.75	0.91	4.24	0.15	0.838	29.0 (95% CI, 11.9–70.3)
The validation cohort								
T-SPOT.TB (positive)	0.63	0.69	0.55	0.75	2.03	0.54	0.669	3.8 (95% CI, 2.0–7.1)
Prediction model (probability ≥ 0.36)	0.72	0.83	0.72	0.83	4.14	0.34	0.801	12.1 (95% CI, 5.9–25.0)
BALF tNGS (positive)	0.66	0.91	0.83	0.79	7.06	0.38	0.782	18.7 (95% CI, 8.0–43.8)
Parallel testing								
BALF tNGS with T-SPOT.TB	0.90	0.64	0.60	0.91	2.46	0.16	0.773	15.0 (95% CI, 5.3–35.9)
BALF tNGS with prediction model	0.94	0.78	0.72	0.96	4.31	0.08	0.847	56.4 (95% CI, 18.7–170.8)

^*a*^ The prediction model includes peripheral blood SFCs of ESAT-6 and CFP-10, BALF concentrations of IL-1β and IL-6, and the BALF CD4/CD8 T-cell ratio.

^*b*^ BALF, bronchoalveolar lavage fluid; tNGS, targeted next-generation sequencing; ROC, receiver operating characteristic.

In patients with a positive blood T-SPOT result, the predicted probability of the model was more than 0.36 in 41/49 (83.7%) SNPT patients and less than 0.36 in 23/36 (63.9%) patients without SNPT. A cut-off predicted probability of 0.36 in the prediction model effectively distinguished SNPT patients from individuals with LTBI in the derivation cohort (OR, 9.1; 95% CI, 3.3–25.1; *P* < 0.001).

### Validation and comparative evaluation in the external cohort

The performance of the prediction model for SNPT was validated in the external cohort using predicted probabilities as classifiers to create ROC curves, yielding an AUC of 0.801 (95% CI, 0.735–0.867). Using a cut-off predicted probability of 0.36, the model achieved a sensitivity of 71.6% (95% CI, 59.9%–81.3%) and a specificity of 82.7% (95% CI, 74.6%–88.7%) for SNPT prediction. Further subgroup analysis showed that the AUC for culture-positive SNPT patients was 0.801 (95% CI, 0.713–0.856), while the AUC for culture-negative SNPT patients was 0.801 (95% CI, 0.722–0.881), with no significant difference between the two groups (*P* = 0.980). Among patients with a positive blood T-SPOT result, the predicted probability of the model was more than 0.36 in 39/42 (92.9%) SNPT patients, and less than 0.36 in 24/34 (70.6%) non-SNPT patients. Thus, a predicted probability above 0.36 effectively differentiated SNPT from LTBI (OR, 31.2; 95% CI, 7.8–124.9; *P* < 0.001). In the five AFB smear-positive TB patients (positive control), the predicted probabilities were 0.99 (IQR, 0.57–1.00).

The diagnostic accuracy of blood T-SPOT, the prediction model, BALF tNGS, and their parallel combinations (i.e., BALF tNGS with T-SPOT, and BALF tNGS with the prediction model) in the validation cohort was presented in the lower part of Table 2. BALF tNGS alone showed a high specificity of 90.7% but a relatively low sensitivity of 65.7%. The prediction model achieved a sensitivity of 71.6% and a specificity of 82.7%, suggesting that it provides additional diagnostic information beyond molecular testing. Parallel testing with BALF tNGS and the prediction model increased sensitivity to 94.0% with a specificity of 78.0%, and showed the strongest association with SNPT (OR, 56.4; 95% CI, 18.7–170.8; *P* < 0.001), outperforming BALF tNGS alone (OR, 18.7; 95% CI, 8.0–43.8), the prediction model alone (OR, 12.1; 95% CI, 5.9–25.0), and the parallel testing with T-SPOT (OR, 15.0; 95% CI, 5.3–35.9). Among all the methods for the detection of active tuberculosis, this combined strategy achieved the highest negative predictive value of 0.96 and an AUC of 0.847, suggesting the complementary diagnostic value of integrating BALF tNGS with the BALF-based immunological prediction model.

## DISCUSSION

In this study, we collected the BALF samples obtained from affected lung segments of SNPT-suspected patients and investigated the cytokine concentrations and lymphocyte responses in these BALF samples. Our data showed that the BALF concentrations of IL-1β and IL-6, together with the BALF CD4/CD8 T-cell ratio, were the independent predictors of SNPT. An immunological model integrating these BALF immune biomarkers with peripheral blood T-SPOT-derived responses provided adjunctive diagnostic information for clinically suspected SNPT and improved diagnostic performance when interpreted in parallel with tNGS.

Although useful for detecting host immune responses to Mtb, the T-SPOT assay is not recommended for diagnosing active TB since blood-based testing cannot distinguish it from LTBI. Lesion-derived samples, such as BALF, can provide more accurate pathological immune profiles, and thus are preferable for immunological diagnosis of active PTB (35). Previous studies have applied the T-SPOT assay to bronchoalveolar lavage mononuclear cells and showed promise in distinguishing SNPT from LTBI (32, 36). However, as an *in vitro* assay of antigen-stimulated effector memory T-cells, it may not reflect real-time immune responses at the lung infection sites, and unstimulated IFN-γ production was frequently observed. In this study, we measured cytokine and lymphocyte responses in lesion-targeted BALF, and found them capable of raising the diagnostic specificity of the blood T-SPOT assay for SNPT, facilitating the discrimination between SNPT and LTBI. In patients with positive blood T-SPOT results, the diagnostic OR of the immunological model covering BALF immune responses was 9.1 (95% CI, 3.3–25.1) in the derivation cohort and 31.2 (95% CI, 7.8–124.9) in the validation cohort, suggesting that BALF immune responses had good discriminatory power between SNPT and LTBI.

In the derivation cohort, the concentrations of IL-1β and IL-6, and the CD4/CD8 T-cell ratio in BALF were retained as independent local immune predictors of SNPT. This finding is broadly consistent with previous BALF immune-profiling studies showing that active TB is accompanied by altered inflammatory cytokine and T-cell responses (31, 32), and our study extends these observations by incorporating a more comprehensive marker panel. Biologically, increased BALF IL-1β may reflect local macrophage-related inflammatory activation during Mtb infection (22), whereas IL-6 is involved in inflammatory amplification and has been linked to TB disease activity and susceptibility (23, 37). The increased BALF CD4/CD8 T-cell ratio further suggests relative enrichment or retention of CD4-dominant T-cell responses within pulmonary lesions, which is compatible with previous evidence linking Mtb-specific CD4^+^ T-cell activation phenotypes to local immune states in SNPT (20, 38). Notably, these differences were observed mainly in lesion-targeted BALF rather than peripheral blood, supporting the view that local immune profiling may better reflect active pulmonary infection than systemic immune measurements (32). By focusing on clinically suspected SNPT, our study extends previous observations in active PTB to a paucibacillary population in which microbiological confirmation is often challenging and lesion-targeted BALF immune features remain insufficiently characterized.

When these local immune predictors were combined with the peripheral blood ESAT-6 and CFP-10 SFCs, the resulting prediction model showed higher sensitivity than BALF tNGS alone in the derivation cohort (73.9% vs 58.0%), whereas BALF tNGS showed higher specificity. This finding is consistent with previous studies suggesting that lung-oriented immunoassays may provide useful adjunctive diagnostic information in SNPT or clinically suspected PTB (39, 40). Furthermore, parallel testing of the prediction model and tNGS substantially improved diagnostic performance over tNGS alone, with higher sensitivity and diagnostic OR in both derivation and validation cohorts. These results support the complementary value of BALF immune biomarkers and molecular assays in the diagnostic assessment of suspected SNPT. Although BALF tNGS detected Mtb-specific sequences in a small proportion of non-SNPT patients, these findings should be interpreted cautiously because molecular positivity alone does not necessarily establish active PTB, and tNGS results may be affected by cross-reactivity or environmental contamination (12, 16). Therefore, BALF tNGS positivity alone was not used to define SNPT in this study, and final classification was based on a composite reference standard integrating clinical, radiological, microbiological, treatment-response, and follow-up evidence.

Culture remains the reference standard for diagnosing pulmonary tuberculosis. However, clinical practice in China demonstrates notably low culture positivity rates for SNPT. Epidemiological data reveal significant regional variations, with only 14.4% culture positivity among SNPT patients in a mid-eastern China study (41), while 32.3% was reported in a Shanghai cohort (11). Our study showed culture positivity rates of around 43% in the derivation and validation cohorts, which are consistent with previous reports. These data suggest that the majority of SNPT cases remain dependent on clinical diagnosis due to limitations in microbiological confirmation. Between culture-positive and culture-negative SNPT patients, we found no significant difference in the demographic characteristics. Although culture-positive patients had significantly higher BALF neutrophil counts in the derivation cohort, lymphocyte counts did not differ. Importantly, the immunological prediction model showed consistent diagnostic accuracy across both groups, indicating good applicability regardless of culture results. To confirm the diagnostic accuracy of the model, a positive control group of culture- and AFB smear-positive patients was included, and their SNPT predicted probabilities by the prediction model were 0.99 (IQR, 0.57–1.00), all above the optimal probability threshold of 0.36.

The limitations of the study are as follows. Firstly, this is an observational study with a relatively small number of SNPT patients and a heterogeneous non-SNPT control group, which may limit the precision of performance estimates and preclude disease-specific subgroup analyses. Secondly, the exploratory comparisons of multiple cytokines and lymphocyte subsets were not formally adjusted for multiple testing, so false-positive findings cannot be completely excluded, although a conservative significance threshold was applied and external validation was performed to support the robustness of the retained markers. Thirdly, the model depends on bronchoscopy and adequate BALF cells, which would limit its applicability in many high-burden TB settings where resources are often limited; therefore, it should be viewed as an adjunctive tool for clinically suspected SNPT patients in whom bronchoscopy is already indicated, rather than a first-line screening test in resource-limited settings (3). Fourthly, the generalizability of the model may be affected by selection bias from enrolling only bronchoscopy-eligible patients, although this bias is at least partially mitigated by the fact that bronchoscopy is routinely performed in smear-negative suspects requiring definitive diagnosis, and by the multicenter external validation. Finally, in high-incidence regions, repeated Mtb exposure, LTBI, and NTM sensitization are more common, which may shape baseline immunity and induce trained immunity, Treg expansion, and cytokine changes, potentially influencing our observed profiles and limiting generalizability to other populations. Further validation in broader SNPT populations is warranted.

In conclusion, our data showed that BALF IL-1β, IL-6, and CD4/CD8 ratio were independent predictors of SNPT. A model combining these biomarkers with peripheral blood T-SPOT-derived ESAT-6 and CFP-10 responses could serve as a valuable adjunct for SNPT diagnosis in clinically suspected non-HIV patients. Although T-SPOT alone showed poor performance, its utility became evident when it was integrated into the BALF-based model. These findings support that the measurement of lesion-targeted BALF immune profiling may facilitate more effective initiation of anti-Mtb therapy.

## Data Availability

Anonymized data and analysis code are available upon reasonable request, subject to ethics committee approval and execution of a data transfer agreement. Public deposition is not permitted due to ethical restrictions and the consent form, but requests to the corresponding author will be responded to within 4 weeks.

## SUPPLEMENTAL MATERIAL

Figure S1 spectrum.03636-25-s0001.docx Representative flow-cytometry pre-gating and lymphocyte-subset identification in PBMC and BALF samples.

Table S1 spectrum.03636-25-s0002.docx Demographic and clinical characteristics of patients in the validation cohort.

ASM does not own the copyrights to Supplemental Material that may be linked to, or accessed through, an article. The authors have granted ASM a non-exclusive, world-wide license to publish the Supplemental Material files. Please contact the corresponding author directly for reuse.
