# Supplementary material for: Diagnostic value of lesion-targeted BALF immune biomarkers combined with T-SPOT.TB for smear-negative pulmonary tuberculosis
Source: Microbiol Spectr. 2026 Sep 1;14(10):e03636-25. doi: 10.1128/spectrum.03636-25 (PMC13689422; doi:10.1128/spectrum.03636-25)
Supplement: Supplementary file 1 [file spectrum.03636-25-s0001.docx]

**Supplementary Figure 1**

**Figure S1.** Representative flow-cytometry pre-gating and lymphocyte-subset identification in PBMC and BALF samples. **A**, Representative initial pre-gating plots for PBMC and BALF samples. Cellular events were initially selected according to FSC-H/SSC-H characteristics, followed by singlet selection using FSC-A versus FSC-H and identification of CD45⁺ low-SSC lymphocytes.

**B**, Representative bivariate plots used for lymphocyte-subset identification in PBMC and BALF samples. In the original analysis workflow, CD4⁺ and CD8⁺ T-cell subsets were defined as CD3⁺CD4⁺CD8⁻ and CD3⁺CD4⁻CD8⁺ cells, respectively. CD4⁺CD29⁺ and CD8⁺CD28⁺ lymphocyte populations were identified using the indicated CD4/CD29 and CD8/CD28 bivariate plots within the gated lymphocyte population. PBMC, peripheral blood mononuclear cells; BALF, bronchoalveolar lavage fluid; FSC, forward scatter; SSC, side scatter.
