# Supplementary material for: Diagnostic value of lesion-targeted BALF immune biomarkers combined with T-SPOT.TB for smear-negative pulmonary tuberculosis
Source: Microbiol Spectr. 2026 Sep 1;14(10):e03636-25. doi: 10.1128/spectrum.03636-25 (PMC13689422; doi:10.1128/spectrum.03636-25)
Supplement: Supplementary file 2 [file spectrum.03636-25-s0002.docx]

**Supplementary Table 1**. Demographic and clinical characteristics of patients in the validation cohort.

|  | **Non-SNPT** | | **SNPT** | | **Non-SNPT**  **vs**  **SNPT** | **Culture-negative SNPT**  **vs**  **Culture-positive SNPT** |
| --- | --- | --- | --- | --- | --- | --- |
|  | non-TB  respiratory illness  *n* = 76 | LTBI + non-TB respiratory illness  *n* = 34 | Culture-negative  SNPT  *n* = 38 | Culture-positive  SNPT  *n* = 29 | *P*-value | *P*-value |
| Gender, male/female (% of male) | 42/34 (55.3) | 21/13 (61.8) | 24/14 (63.2) | 13/16 (44.8) | 0.790 | 0.135 |
| Age in years, mean (SD) | 62.9 (12.6) | 55.6 (17.6) | 54.5 (15.2) | 49.7 (18.2) | 0.011 | 0.154 |
| BMI, kg/m^2^ (SD) | 22.6 (3.4) | 24.0 (2.9) | 21.9 (3.1) | 21.7 (2.3) | 0.019 | 0.556 |
| Diabetes mellitus, no. (%) | 9 (11.8) | 2 (5.9) | 8 (21.1) | 3 (10.3) | 0.209 | 0.401 |
| Symptoms: |  |  |  |  |  |  |
| Fever, no. (%) | 6 (7.9) | 4 (11.8) | 5 (13.2) | 8 (27.6) | 0.048 | 0.139 |
| Cough, no. (%) | 59 (77.6) | 18 (52.9) | 22 (57.9) | 21 (72.4) | 0.507 | 0.219 |
| Coughing up blood or mucus, no. (%) | 33 (43.4) | 10 (29.4) | 14 (36.8) | 18 (62.1) | 0.276 | 0.041 |
| Chest pain, no. (%) | 2 (2.6) | 4 (11.8) | 5 (13.2) | 5 (17.2) | 0.055 | 0.905 |
| Night sweats, no. (%) | 3 (3.9) | 0 (0.0) | 0 (0.0) | 2 (6.9) | 0.920 | 0.184 |
| Tiredness, no. (%) | 4 (5.3) | 0 (0.0) | 2 (5.3) | 2 (6.9) | 0.725 | 0.781 |
| Peripheral blood cell count (10^9/L): |  |  |  |  |  |  |
| Leukocyte number, median (IQR) | 6.7 (5.8–8.0) | 6.4 (5.2–7.6) | 6.6 (5.4–8.9) | 6.7 (4.7–7.6) | 0.585 | 0.397 |
| Neutrophil number, median (IQR) | 4.0 (3.1–5.2) | 4.0 (2.9–5.0) | 4.7 (3.3–6.0) | 3.9 (2.7–5.0) | 0.632 | 0.220 |
| Lymphocyte number, median (IQR) | 1.7 (1.3–2.1) | 1.8 (1.2–2.1) | 1.5 (1.1–1.9) | 1.5 (1.0–1.9) | **0.004** | 0.976 |
| Monocyte number, median (IQR) | 0.5 (0.4–0.7) | 0.5 (0.4–0.6) | 0.5 (0.4–0.7) | 0.4 (0.3–0.7) | 0.510 | 0.291 |
| T-SPOT.TB |  |  |  |  |  |  |
| Positivity rates, no. (%) | 0 (0.0) | 34 (100.0) | 22 (57.9) | 20 (69.0) | **< 0.001** | 0.353 |
| SFC for ESAT-6 (/10⁶ PBMCs), median (IQR) | 0.0 (0.0–1.0) | 10.0 (4.8–26.3) | 5.0 (0.0–15.5) | 8.0 (3.0–17.0) | **< 0.001** | 0.392 |
| SFC for CFP-10 (/10⁶ PBMCs), median (IQR) | 0.0 (0.0–0.0) | 6.5 (2.0–22.0) | 7.5 (0.3–23.8) | 8.0 (1.0–37.0) | **< 0.001** | 0.700 |
| BALF cell count (10^6/L): |  |  |  |  |  |  |
| Nucleated cell number, median (IQR) | 135.0 (52.5–317.8) | 105 (40.0–337.5) | 380.0 (91.0–770.0) | 300.0 (122.5–975.0) | **< 0.001** | 0.489 |
| Neutrophil number, median (IQR) | 18.4 (4.2–96.8) | 5.6 (1.3–32.0) | 18.0 (2.3–439.0) | 45.9 (6.7–772.9) | 0.043 | 0.539 |
| Lymphocyte number, median (IQR) | 7.2 (2.4–30.0) | 7.2 (1.8–40.5) | 23.8 (3.4–95.2) | 15 (4.3–39.2) | **0.009** | 0.454 |
| Macrophage number, median (IQR) | 49.4 (18.0–136.5) | 49.6 (13.1–145.0) | 102.3 (43.5–200.5) | 105.8 (67.2–226.6) | **0.001** | 0.514 |
| Radiological manifestation: |  |  |  |  |  |  |
| Extent of lung lesions |  |  |  |  |  |  |
| < 25%, no. (%) | 53 (69.7) | 29 (85.3) | 21 (55.3) | 13 (44.8) | **0.002** | 0.336 |
| 25-50%, no. (%) | 21 (27.6) | 5 (14.7) | 11 (28.9) | 13 (44.8) | 0.052 | 0.223 |
| > 50%, no. (%) | 2 (2.6) | 0 (0.0) | 6 (15.8) | 3 (10.3) | **0.002** | 0.683 |
| Cavity, no. (%) | 3 (4.0) | 1 (2.9) | 6 (15.8) | 7 (24.1) | **0.001** | 0.433 |
| Bilateral disease, no. (%) | 59 (77.6) | 21 (61.8) | 20 (52.6) | 17 (58.6) | 0.023 | 0.513 |
| Positivity rates of culture for Mtb, no. (%) | 0 (0.0) | 0 (0.0) | 0 (0.0) | 29 (100.0) | ND | ND |
| Positivity rates of tNGS for Mtb, no. (%) | 6 (7.9) | 3 (8.8) | 21 (55.3) | 23 (79.3) | **< 0.001** | 0.040 |

*Note:* The data were compared by chi-square test, except that age was compared by unpaired *t*-test, and BMI, peripheral blood cell analysis, and BALF cell analysis were compared by Mann-Whitney U test. Statistically significant differences (*P* < 0.01) are indicated by bold text.

*Abbreviation:* LTBI, latent tuberculosis infection; SNPT, smear-negative pulmonary tuberculosis; SD, standard deviation; BMI, body mass index; IQR, interquartile range; CFP-10, culture filtrate protein 10; ESAT-6, early secreted antigenic target 6; PBMC, peripheral blood mononuclear cells; Mtb, Mycobacterium tuberculosis; BALF, bronchoalveolar lavage fluid; tNGS, targeted next-generation sequencing; ND, not determined.
